# Safety and effectiveness of self-managed medication abortion provided using online telemedicine in the United States: A population based study

**DOI:** 10.1016/j.lana.2022.100200

**Published:** 2022-02-17

**Authors:** Abigail R.A. Aiken, Evdokia P. Romanova, Julia R. Morber, Rebecca Gomperts

**Affiliations:** a LBJ School of Public Affairs, University of Texas at Austin, Austin, TX 78713, USA; b Aid Access, Amsterdam, the Netherland; c Vrije Universiteit, Amsterdam, the Netherland

**Keywords:** Abortion, Self-managed, Online telemedicine, United States

## Abstract

**Background:**

As access to clinical abortion care becomes increasingly restricted in the United States, the need for self-managed abortions (i.e. abortions taking place outside of the formal healthcare setting) may increase. We examine the safety, effectiveness, and acceptability of self-managed medication abortion provided using online telemedicine.

**Methods:**

We retrospectively examined records of the outcomes of abortions provided by the sole online telemedicine service providing self-managed medication abortion in the U.S. We calculated the prevalence of successful medication abortion (the proportion who ended their pregnancy without surgical intervention); the prevalence of serious adverse events (the proportions who received intravenous antibiotics and blood transfusion); and assessed whether any deaths were reported to the service. We also examined the proportions who were satisfied and felt self-management was the right choice.

**Findings:**

Between March 20th 2018 and March 20th 2019, abortion medications were mailed to 4,584 people and 3,186 (70%) provided follow-up information. Among these, 2,797 (88%) confirmed use of the medications and provided outcome information, while 389 (12%) confirmed non-use. Overall, 96.4% (95% CI 95.7% to 97.1%) of those who used the medications reported successfully ending their pregnancy without surgical intervention and 1.0% (CI 0.7%-1.5%) reported treatment for any serious adverse event. Among these, 0.6% (CI 0.4% to 1.0%) reported receiving a blood transfusion, and 0.5% (CI 0.3% to 0.9%) reported receiving intravenous antibiotics. No deaths were reported to the service by family, friends, the authorities, or the media. Among 2,268 who provided information about their experience, 98.4% were satisfied and 95.5% felt self-management was the right choice.

**Interpretation:**

Self-managed medication abortion provided using online telemedicine can be highly effective with low rates of serious adverse events. In light of increasingly restricted access to in-clinic abortion in the U.S., it may offer a safe and effective option for those who cannot access clinical care.

**Funding:**

The Society of Family Planning and The National Institutes of Health.

**Research in context**

**Evidence before this study**

In many U.S. states, access to abortion in the clinic setting is becoming increasingly restricted. In the face of dwindling access, there is evidence that people in the United States are self-managing their abortions (i.e. sourcing and conducting them outside of the formal healthcare setting). While self-managed abortions have been happening in North America for centuries using botanicals, and later misoprostol, one important contemporary method of self-management is the use of online telemedicine to provide the abortion medications mifepristone and misoprostol. No studies, however, have examined the safety, effectiveness, and acceptability of self-managed medication abortion using this model in the United States. We conducted a systematic search of MEDLINE and PubMed using the MeSH terms ‘Abortion’, ‘Self-managed’, ‘United States’, ‘Safety’ and ‘Effectiveness’ with no language or date restrictions, and found no relevant reports as of September 2021. Using data from the sole online telemedicine organization that provides self-managed medication abortion to people in the United States, we provide here an analysis of the reported outcomes of self-managed abortions provided between March 20th 2018 and March 20th 2019. We report both the proportion that resulted in successful termination of pregnancy and the proportion that resulted in serious adverse events. We also describe the acceptability of these self-managed abortions based on user experience.

**Added value of this study**

This study presents the first evidence that self-managed medication abortion provided through online telemedicine in the United States is effective, acceptable to users, and has a very low rate of serious adverse outcomes. This evidence adds to a global body of data showing that self-managed medication abortion is safe and effective. In this study, 70% of those to whom medications were shipped provided follow-up information about their abortions, and among these 2,797 (88%) confirmed use of the medications and provided outcome information, while 389 (12%) confirmed non-use. Among those who used the medications, 96% were able to end their pregnancies without surgical intervention from a clinical provider, a rate comparable with the in-clinic setting. Treatment for serious adverse events was uncommon with 1% receiving a blood transfusion or intravenous antibiotics. No deaths were reported to the service by family, friends, the authorities, or the media. Among the 2,268 people who provided information about their experience, 98% expressed satisfaction with their abortion experience and 96% said it was the right choice.

**Implications of all the available evidence**

As restrictive abortion legislation continues to create substantial barriers to in-clinic abortion in many U.S. states, abortion self-managed outside of the formal healthcare setting is an important option for those who cannot or prefer not to access clinical care. This study provides key evidence for clinicians, policymakers, and the public that safe, effective, self-managed medication abortion is available in the United States. However, people who self-manage are still vulnerable to legal risks and in some states unjust prosecutions have occurred. Rather than being subject to criminalization, self-managed abortion should be a legitimate part of a spectrum of options for abortion care that must be made available in the United States.

Alt-text: Unlabelled box

## Introduction

Abortion access is approaching cross-roads in the United States. The Supreme Court is currently considering an enacted Mississippi law that bans abortion at 15 weeks’ gestation^1^ and recently allowed a 6-week ban in effect in Texas to stand.^2^ Although the right to choose established by *Roe v. Wade* in 1973 still currently stands, access to abortion in the clinic setting is moving further out of reach due to restrictive state legislation. One hundred and eight abortion restrictions were enacted in state legislatures in 2021, more than any other year since *Roe*.^3^ These laws make clinical abortion harder to access by imposing waiting periods and medically unnecessary medical tests on patients and requiring clinics and providers to conform to unnecessary administrative regulations.^3^

Recent research suggests that one possible consequence of increasing barriers to in-clinic abortion is that more people will self-manage.^4^^,^^5^ A self-managed abortion is one that takes place outside of the formal healthcare setting, and includes a spectrum of methods, such as the abortion pills mifepristone and/or misoprostol, menstrual extraction, botanicals, herbs, vitamins, beverages, and ingestion of toxic substances and physical injury. Self-managed abortion has been happening in North America for centuries and recent estimates suggest that approximately 7 percent of U.S. women have attempted a self-managed abortion in their lifetime.^6^

An important consideration for people who self-manage is the safety and effectiveness of the method they are using. Since 2018, self-managed abortion using mifepristone and misoprostol has been available through online telemedicine in the U.S. via a non-profit service called Aid Access.^7^ The service received 57,506 requests from people in the U.S. in its first two years of operation.^4^ While clinic-based and physician-led telemedicine models that provide medication abortion within the formal healthcare setting are also available in some U.S. states,^8^, ^9^, ^10^ Aid Access is distinct from these service models because it offers self-managed abortion, operating outside of the formal U.S. healthcare setting in all 50 states. Provision of self-managed medication abortion using similar services, such as Women on Web and Women Help Women, has been explored in other countries,^11^, ^12^, ^13^ but outcomes have not been studied in any U.S.-based population. Using data from Aid Access, the objective of this study is to examine the safety, effectiveness, and acceptability of self-managed medication abortion provided via online telemedicine in the U.S.

## Methods

### Data

Aid Access currently provides medication abortion up to 10 weeks gestation at the time of request, which is made using an online consultation form. A doctor reviews the form to ensure no contraindications and provides a prescription of 200mg mifepristone to be taken orally and 800mcg misoprostol to be taken sub-lingually, along with an additional 800mcg of misoprostol for use if needed, according to the World Health Organization (WHO) recommended dosage regimen for medication abortion.^14^ A partner organization then mails the medications along with usage instructions. A donation of $110 to support the service is requested, but those who cannot afford it are asked to donate what they can. An online non-clinical helpdesk team is available to answer questions. Four weeks after receipt of the medications, users are invited to report their abortion outcomes using an online evaluation form or via an email to the helpdesk.

Our dataset includes all U.S. residents to whom abortion medications were shipped between March 20th 2018 and March 20th 2019. Since Aid Access is the sole organization of its kind serving the U.S., our sample represents the universe of people in the U.S. self-managing a medication abortion using online telemedicine outside the formal healthcare system. De-identified data from the online consultation form, follow-up form, and emails were provided by Aid Access. All individuals in the sample consented to the anonymized use of their data at the aggregate level for research purposes.

The online consultation form includes self-reported information about age, weeks’ gestation, parity, feelings about the decision to have an abortion, any medical contraindications, whether or not a person has had an ultrasound scan for the current pregnancy, knows someone who can be with them during their abortion, and lives within 60 min of a hospital. We categorized age as “Under 20 years”, “20–24 years” and into 5-year increments thereafter, with a final group of “40 years and over”. Gestation was reported as “< 7 weeks” or “7–10 weeks”, which represents gestation at the time of the consultation. Those who did not have an ultrasound scan used a pregnancy calculator based on their last menstrual period. Number of children was reported numerically, and we constructed categories of “0” and “1 or more”. Feelings about the decision to have an abortion were reported as “I can cope with my feelings regarding my decision” and “I have some worries about my decision and would like further information”. Those who expressed worries were directed to appropriate sources of information. Medical history questions included the presence of any contraindications (e.g. bleeding disorders, inherited porphyrias, allergies to mifepristone or misoprostol) or medical conditions that required additional medical screening (e.g. having an IUD in place or having a suspected STI).

The evaluation form is based on similar follow-up instruments used in the clinical setting and is sent to participants 4 weeks after receipt of the medication. Available information included the number who confirmed delivery of the medications, the number who confirmed whether or not they used the medications, and the outcome of the pregnancy or abortion. Those who confirmed using the medications were asked about gestation at the time of use, whether or not they were still pregnant, whether or not they received any clinical intervention to help end the pregnancy (Dilation and Curettage (D&C) or vacuum aspiration), and any other treatment they received for a possible serious adverse event following their abortion. Those who confirmed not using the medications were asked about the outcome of their pregnancy.

### Analysis

We compared available clinical and demographic characteristics among those who provided follow-up information and those who did not. We conducted chi-squared difference of proportions tests using an alpha level of 0.05 to indicate statistical significance to check for any systematic differences between the two groups that might affect the outcome of their abortions.

Among those for whom self-reported information on outcomes was available, we examined these in the overall sample as well as constructing two groups: those reported a gestation of 10 weeks or fewer at the time of using the pills, and those who reported a gestation of over 10 weeks. This threshold was chosen to allow comparison with outcomes of medication abortion in the clinic setting, where the mifepristone-misoprostol combined regimen is approved by the FDA through 70 days gestation.^15^ While requestors must be 10 weeks pregnant or less at the time of filling out the consultation form, the medications may take 1–3 weeks to arrive and thus some individuals may be over 10 weeks at the time of use. We first examined the proportion who reported that they were no longer pregnant, and then the proportion for whom medication abortion was successful according to the standard definition of success in the Medical Abortion Reporting of Efficacy (MARE) Guidelines, *i.e.* the proportion who were able to expel their pregnancy without the need for surgical intervention.^16^ Next, we examined the prevalence of reported serious adverse events, following to the extent possible the categories defined by Cleland et al.^17^ Information was available on receipt of IV antibiotics and blood transfusion and we also assessed whether any deaths were reported, recognizing that we are relying on reporting by friends, family members, the authorities, or the media. We calculated point-estimates and exact binomial 95% confidence intervals (CI) both for the overall population and for the binary gestation categories available in our dataset. To compare outcomes between the two gestation groups we used Fisher's exact test and considered findings statistically significant at an alpha level of 0.05.

The follow-up form also included a series of “yes/no” questions asking about the abortion experience, including satisfaction with the service, and whether: using it had been the right choice; affording the full donation (which at the time of data collection was $90) had been difficult; enough information had been provided about the abortion process; and enough support was available from family and friends. We calculated the proportions of people answering “yes” and “no” to each question.

Data analysis was conducted using Stata version 15.1.^18^ The Institutional Review Board of the University of Texas at Austin approved the study.

### Role of the funding source

Neither funding source that supported the investigators during the study had any involvement in study design, data collection, analysis or interpretation, and had no role in the writing of this manuscript or the decision to submit for publication.

## Results

**Figure 1:**
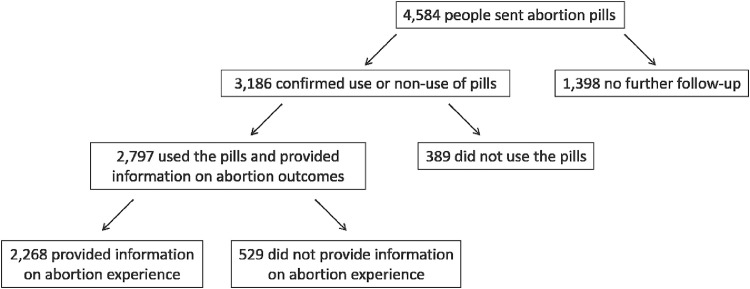
U.S. residents accessing abortion medications through Aid Access.

Between 20th March 2018 and 20th March 2019, Aid Access provided mifepristone and misoprostol by mail to 4584 people. Among these, 3,186 provided follow-up information for a follow-up rate of 70% (Figure 1). Of those who provided follow-up information, 2797 (88%) confirmed use of the medications and provided information on the outcome of their abortion, while 389 (12%) confirmed non-use of the medications. Reasons for non-use included spontaneous pregnancy loss (45%), accessing abortion care in a clinic (21%), deciding to continue the pregnancy (19%), shipping delays (6%), having self-managed using another method (3%), the pregnancy being a false alarm (3%); and experiencing symptoms of an ectopic pregnancy, for which they reported receiving clinical treatment (0.5%). An additional 3% did not specify a reason for not using the medications.

The demographic and clinical characteristics of those who provided follow-up information vs. those who did not are shown in Table 1. Among those who provided follow-up, 94.9% reported being under 7 weeks pregnant at the time of requesting the service. The majority (63.1%) were aged under 30. Virtually all (99.4%) felt OK about their decision to have an abortion and none had any contraindications to abortion medications. A greater proportion of those who provided follow-up were aged 20 or over (89.1% vs. 85.4%, *p* < 0.01), already had children (58.1% vs. 48.4%, *p* < 0.001), and had not received an ultrasound scan prior to their abortion (90.4% vs. 87. 6%, *p*=0.005). There were no significant differences in any characteristic, including gestation, that might reasonably bias the follow-up group towards more favorable outcomes.

**Table 1:** Demographic and clinical characteristics of those to whom abortion medications were provided by Aid Access (*N*=4584).

Characteristic	Provided follow-up information (*N*=3,186)	Did not provide follow-up information (*N*=1,398)	*P*-value
Gestation (weeks)			
7 weeks or fewer	3,025 (94.9)	1,337 (95.6)	0.317
8-10 weeks	161 (5.1)	61 (4.4)	
Age (years)			
Under 20	347 (10.9)	206 (14.7)	0.005
20-24	795 (25.0)	357 (25.5)	
25-29	867 (27.2)	359 (25.7)	
30-34	661 (20.7)	269 (19.2)	
35-39	376 (11.8)	152 (10.9)	
40 and over	140 (4.4)	55 (3.9)	
Children			
0	1,335 (41.9)	721 (51.6)	<0.001
1 or more	1,851 (58.1)	677 (48.4)	
Feelings about abortion decision			
Ok with decision	3,167 (99.4)	1,386 (99.1)	0.317
Troubled by decision	19 (0.6)	12 (0.9)	
Contraindications to medication abortion			
Yes	0 (0.0)	0 (0.0)	–
No	3,186 (100.0)	1,398 (100.0)	
Ultrasound Scan			
Yes	305 (9.6)	172 (12.4)	0.005
No	2,881 (90.4)	1,226 (87.6)	
Current STI			
Yes	14 (0.4)	9 (0.6)	0.367
No	3,172 (99.6)	1,389 (99.4)	
IUD in place			
Yes	6 (0.2)	3 (0.2)	0.853
No	3,180 (99.8)	1,395 (99.8)	
Knows somebody who can be present during the abortion			
Yes	3,111 (97.6)	1,363 (97.5)	0.761
No	75 (2.4)	35 (2.5)	
Within 60 mins of a hospital			
Yes	3,139 (98.5)	1,369 (97.9)	0.144
No	47 (1.5)	29 (2.1)	

Among those who confirmed use of the medications (*n*=2,797), 2402 (86%) reported being under 10 weeks pregnant, while 395 (14%) reported being 10 weeks pregnant or more. Overall, 99.0% of all those who used the medications reported having ended their pregnancies (Table 2), and 96.4% reported a successful medication abortion (i.e., ending their pregnancies with no surgical intervention). There was no significant difference by gestation in the proportion reporting ending their pregnancies (99.1% vs. 98.2%, *p*=0.097), but those who were less than 10 weeks pregnant had a lower rate of surgical intervention compared to those who were 10 weeks or more (2.0% vs. 6.1%, *p* < 0.001). Overall, among the 72 people who reported receiving a procedure to help end their pregnancy, 54 received D&C, 12 received aspiration, and 6 did not specify procedure type. No ectopic pregnancies were reported among those who confirmed use of the medications.

**Table 2:** Outcome of abortion reported by people who self-managed a medication abortion using Aid Access (*N*=2797).

Outcome	All gestations (*N*=2797)Frequency (percent, 95% CI)	Up to 10 weeks (*N*=2402)Frequency (percent, 95% CI)	Over 10 weeks (*N*=395)Frequency (percent, 95% CI)	*P*-value
Pregnancy				
No longer pregnant	2,769 (99.0, 98.6-99.3)	2,381 (99.1, 98.7-99.5)	388 (98.2, 96.4-99.3)	0.097
Surgical Intervention				
Reported surgical intervention	72 (2.6, 2.0-3.2)	48 (2.0, 1.5-2.6)	24 (6.1, 3.9-8.9)	<0.001
Successful medication abortion				
No longer pregnant and no surgical intervention	2,697 (96.4, 95.7-97.1)	2,333 (97.1, 96.4-97.8)	364 (92.1, 89.1-94.6)	<0.001

Potentially serious adverse events were not common (Table 3). Overall, 29 people (1.0%) reported experiencing any serious adverse event. Of these, 18 people (0.6%) reported receiving a blood transfusion and 15 (0.5%) reported receiving IV antibiotics (4 people reported receiving both). No deaths were reported by family, friends, clinicians, the authorities, or the media. Rates of adverse events overall were more common among those who reported a gestation of 10 weeks or more as compared with those who were less than 10 weeks (2.3% vs. 0.8%, *p*=0.009).

**Table 3:** Serious adverse events reported by people who self-managed a medication abortion through Aid Access (*N*=2797).

Outcome	All gestations (*N*=2797)Frequency (percent, 95% CI)	Up to 10 weeks (*N*=2402)Frequency (percent, 95% CI)	Over 10 weeks (*N*=395)Frequency (percent, 95% CI)	*P*-value
Serious Adverse Event				
Blood transfusion	18 (0.6, 0.4-1.0)	11 (0.5, 0.2-0.8)	7 (1.8, 0.7-3.6)	0.002
IV Antibiotics	15 (0.5, 0.3-0.9)	12 (0.5, 0.3-0.9)	3 (0.8, 0.2-2.2)	0.509
Death	0 (0.0, 0.0-0.0)	0 (0.0, 0.0-0.0)	0 (0.0, 0.0-0.0)	–
Any serious adverse event*	29 (1.0, 0.7-1.5)	20 (0.8, 0.5-1.3)	9 (2.3, 1.1-4.3)	0.009

⁎ Each serious adverse event is counted only once.

Among the 2797 people who provided follow-up information, 2268 (81%) reported on the acceptability of their self-management experience (Table 4). Almost all (98.4%) felt satisfied with the service, and 95.5% felt it was the right choice for them. Most (98.1%) felt they had enough information on how to use the medications, and 93.4% felt they had enough information on what to expect from the process. Fewer (81%) felt that they had enough support from family or friends, and 61.8% had difficulty affording the full requested donation.

**Table 4:** Experiences reported by people who self-managed a medication abortion through Aid Access (*N*=2268).

Outcome	All gestations (*N*=2268)* Frequency (percent)
Felt satisfied with the service	2,231 (98.4)
Felt self-management using the service was the right choice	2,166 (95.5)
Felt had enough information on how to use pills	2,225 (98.1)
Felt had enough information on what to expect to see and feel	2,118 (93.4)
Felt had enough support from family/friends	1,837 (81.0)
Had difficulty affording the full requested donation	1,401 (61.8)

⁎ 529 people who confirmed using medications did not provide information on acceptability outcomes.

## Discussion

We used a data set containing all available outcomes of self-managed medication abortions provided through online telemedicine, outside the formal healthcare system, in the U.S. for one year. We found that abortions self-managed using this model were highly effective, with reported success rates comparing favorably with medication abortions carried out up to 10 weeks within the formal U.S. healthcare setting.^19^ The reported prevalence of serious adverse events was very low, and the users of service reported high levels of satisfaction.

Our results offer the first insight into the outcomes of self-managed medication abortions provided using online telemedicine in the U.S. The high effectiveness rates and low prevalence of serious adverse events we found mirror findings from other countries where medication self-management is used.^20^, ^21^, ^22^ We note that although most people in our study did not receive an ultrasound, they reported awareness of the duration of their pregnancy at the time of medication use. These findings are in line with prior evidence suggesting that last menstrual period is an accurate method for determining gestation in early pregnancy^23^ and WHO guidelines, which clearly specifies that ultrasound is not a necessary pre-requisite for medication abortion.^14^

Our findings also add to evidence on the safety and effectiveness of self-managed medication abortion beyond 10 weeks. While rates of surgical intervention were higher among the small proportion in our study who used the medications after 10 weeks compared to those at 10 weeks or under, almost all were able to end their pregnancies and the rate of successful medication abortion was similar to other studies examining medication abortion in the late first trimester,^24^ and medication self-management after 13 weeks using an accompaniment model.^25^ Those self-managing after 10 weeks in our study also tended to have experienced shipping delays and some received modified instructions according to the WHO protocol for medication abortion at 12 weeks and over^14^ and additional support from Aid Access. In addition, no ectopic pregnancies were reported after using the medications and indeed a small number were diagnosed quickly by the service at the time of initial contact.

This study has several limitations. The first is that abortion outcomes were self-reported by people who self-managed. However, since these abortions take place outside of the formal healthcare system, self-reporting is by definition the only possible method of follow-up. Moreover, a previous large randomized controlled trial showed that self-assessment of the outcome of medication abortion was non-inferior to clinical follow-up, indicating that people are capable of determining on their own whether or not their abortion has been successful.^26^ Since an examination of the outcomes of medication abortions taking place outside the formal healthcare setting cannot be dealt with by a randomized controlled trial or clinical trial, we have drawn on the best available “real world” data to answer these important questions.

Second, although the 70% follow-up rate in this study is incomplete, it is on par with or better than many clinical studies, since most outcomes are only recorded if patients decide to follow up with the clinic.^27^ Moreover, we took a more conversative approach than most clinical studies in that we did not consider those for whom no outcome data were available as presumed successful abortions. Third, while self-reporting could be subject to recall or social desirability bias, the short time period between the abortion and the collection of follow-up information should minimize recall bias.

While treatment for serious adverse events was a rarely reported outcome in our study, rates were still higher than those reported in studies of abortions taking place in the clinical setting.^19^^,^^28^ We note, however, that we are not able to verify whether the treatment received by users of the service who did engage with the formal healthcare system was appropriate to their situation. For example, most of those who reported receiving surgical intervention also received antibiotics, which may have been given prophylactically or to treat an existing infection. Studies in other countries have shown that rates of intervention and additional treatment during clinical abortion follow-up are highly variable, especially in countries where hospital staff are not trained to care for abortion patients.^29^ While we did examine whether any deaths were reported to the service, we were of course unable to fully assess whether any deaths occurred in the group that did not provide follow-up information.

It is also important to consider the rates of self-reported adverse events shown in this study in the context of the other possible outcomes for people in the U.S. who cannot access in-clinic abortion care. Between 200,000 and 1.2 million unsafe abortions are estimated to have taken place per year in the U.S. in the 1950s and 1960s pre-*Roe*, with the resulting burden of morbidity and mortality falling disproportionately on racially minoritised people.^30^ While we do not expect the same prevalence of unsafe abortion today, we cannot assume that none will occur. Moreover, those forced to carry a pregnancy to term would also be at higher risk than those who self-manage using Aid Access. Rates of hemorrhage postpartum in the U.S. are over five times higher than those reported in this study, with Black women at disproportionately high risk.^31^^,^^32^

The trajectory of highly restricted access to in-clinic abortion in the U.S. and the future possibility of full abortion bans in some states means that self-managed abortion is likely to become an increasingly used alternative. The FDA recently permanently suspended the in-person dispensing requirement on mifepristone, paving the way for expanded clinic-based telemedicine abortion services. However, at least 19 states already have laws banning telemedicine for medication abortion and in these jurisdictions the FDA ruling will have little effect.^33^ These states are the also the ones with the highest rates of requests to Aid Access.^4^ In the majority of U.S. states, self-management is not illegal, and clinicians who may provide follow-up care have no reporting obligation.^34^ It is important to note, however, that there have been 24 cases of unjust prosecution for alleged self-management since 2000, with populations already subject to increased surveillance and biased treatment, including people with low incomes and racially minoritised people, at highest risk.^35^ While legal risks remain, our findings demonstrate that self-management using medications provided through online telemedicine is a safe, effective, and acceptable option for people in the U.S. and thus it is both an important method of harm reduction and a means of preserving reproductive autonomy.

## Contributors

ARAA conceived of the original research question. All authors contributed to the study design. RG, ER, and JM compiled and provided the de-identified Aid Access data. ARAA conducted the statistical analyses and prepared the tables and figures. ARAA and RG did the initial data interpretation. ARAA, wrote the first draft of the manuscript. All authors contributed to final data interpretation and contributed to and approved the final draft of the manuscript. ARAA and RG verified the underlying data. All authors had full access to all the data and all authors accept responsibility to submit the manuscript for publication. No medical writers were involved in the preparation of the manuscript.

## Data sharing statement

Authors are not able to directly provide access to data. Any direct requests submitted to Aid Access will be dealt with according to their internal policies and procedures.

## Declaration of interests

ARAA reports grant support from the Society of Family Planning (Grant # SFPRF12-MA1) and received infrastructure support from the National Institutes of Health (Grant # P2CHD042849). ARAA is a Council Member of the British Society of Abortion Care Providers (BSACP). RG is the Founder and Director of Aid Access, ED is a helpdesk coordinator at Aid Access, and JM interned at Aid Access during her program of study at VU.
